# Improving self-care, HbA1c control, and quality of life in type 2 diabetes: a self-regulation intervention based on the individual and family self-management theory

**DOI:** 10.3389/fcdhc.2025.1712334

**Published:** 2026-01-07

**Authors:** Fadli Fadli, Nursalam Nursalam, Elly Lilianty Sjattar, Nilawati Uly

**Affiliations:** 1 Doctoral Student of Nursing, Faculty of Nursing, Universitas Airlangga, Surabaya, Indonesia; 2 Faculty of Health, Mega Buana University, Palopo, Indonesia; 3 Nursing Department, Faculty of Nursing, Universitas Airlangga, Surabaya, Indonesia; 4 Nursing Department, Faculty of Nursing, Hasanuddin University, Makassar, Indonesia

**Keywords:** HbA1c, IFSMT, quality of life, self-care, self-regulation, type 2 diabetes mellitus

## Abstract

**Background:**

Type 2 diabetes mellitus (T2DM) remains a global health challenge, often associated with poor self-care, inadequate glycemic control, and reduced quality of life. Conventional diabetes education mainly focuses on biomedical aspects, with limited attention to psychological self-regulation and family support, resulting in suboptimal outcomes.

**Aim:**

This study aimed to evaluate the effectiveness of a self-regulation intervention based on the *Individual and Family Self-Management Theory* (IFSMT) in improving self-care ability, Glycemic control (HbA1c), and quality of life in patients with type 2 diabetes mellitus (T2DM).

**Method:**

A quasi-experimental pretest–posttest design with a control group was used to evaluate 90 patients with T2DM recruited from primary health centers. Participants were randomly assigned to either the intervention or control groups. The intervention group received a 12-week IFSMT-based self-regulation module, while the control group received standard diabetes education. Outcomes measured included self-care ability (knowledge, belief, skills, stress management), HbA1c levels, and quality of life. Data were analyzed using chi-square, *Wilcoxon Signed Rank Test*, and *the Mann-Whitney U Test* statistical tests with a significance level of p < 0.05.

**Result:**

The intervention group showed significant improvements in self-care ability (p < 0.001) and quality of life (p < 0.01), as well as a significant reduction in HbA1c levels (p < 0.05) compared with the control group. These findings suggest that applying self-regulation principles can enhance diabetes management beyond conventional education.

**Conclusion:**

The IFSMT-based self-regulation intervention was effective in improving self-care, glycemic control, and quality of life in patients with type 2 diabetes mellitus (T2DM). Integration of this intervention into nursing practice and diabetes education programs may strengthen long-term management and reduce the risk of complications.

## 1 Introduction

Type 2 diabetes mellitus (DMT2) is a major global health problem characterized by a continuously increasing prevalence and substantial complications that negatively affect patients’ quality of life (1, 2). In 2021, an estimated 537 million adults (10.5% of the population aged 20–79 years) were living with diabetes, and this number is projected to rise to 783 million by 2045 (3). DMT2 is the focus of this study because it represents the vast majority of diabetes cases and is strongly influenced by modifiable self-care behaviors, making it particularly suitable for self-regulation and family-based interventions. Glycemic control, commonly measured through HbA1c levels (4), plays a critical role in preventing microvascular and macrovascular complications (5).

Despite the availability of treatment and education, many individuals with type 2 diabetes continue to face challenges in engaging in essential self-care behaviors such as healthy dietary practices, regular physical activity, psychosocial management, and consistent medication use, which are critical for maintaining stable blood glucose levels (2, 6). These challenges are reflected in the proportion of patients who experience difficulties in maintaining healthy dietary patterns (43.7%), engage in sufficient physical activity (40%), monitor blood glucose levels (31.9%), adhere to treatment schedules (22.2%), or perform adequate foot care (22.72%). Uncertain and inconsistent self-care patterns contribute to poor glycemic control and increase the risk of long-term complications (7, 8).

If glycemic control is not achieved, DMT2 can lead to serious macrovascular and microvascular complications such as diabetic kidney disease, retinopathy, and neuropathy (9, 10), which significantly impair patients’ quality of life (11) and remain a leading cause of morbidity and mortality (12). Consistent self-care is therefore a key determinant of metabolic control (13). Evidence shows that consistent medication use, healthy dietary practices, and regular physical activity are associated with lower HbA1c levels. (14–16) and better quality of life outcomes (17–20). However, sustaining these behaviors remains challenging due to psychological factors, fluctuating motivation, and limited social support (21, 22).

Family support has been recognized as one of the most influential factors in strengthening patients’ engagement in self-care activities, enhancing coping strategies, and improving overall diabetes management (23–25). Evidence also shows that family involvement can strengthen patients’ engagement in medication management and healthy dietary practices (26) and enhance the effects of individual self-management strategies. Several studies have reported that family-based programs yield better outcomes than standard routine care in improving diabetes self-management (27, 28). Within the Individual and Family Self-Management Theory (IFSMT) framework, these findings reflect the interaction between individual and family processes, where supportive family environments reinforce self-regulation and self-care behaviors. However, many existing interventions remain limited to general education without a systematic theoretical foundation, resulting in inconsistent effects on HbA1c and quality of life (29, 30).

However, changes in individual factors alone are often insufficient to sustain long-term diabetes self-management. Daily behaviors such as dietary choices, physical activity, and stress regulation occur within the family environment, where emotional, instrumental, and behavioral support strongly influence patients’ consistency in maintaining self-care (31). Therefore, a family-focused approach is essential for achieving sustainable behavior change, particularly in cultural contexts where family involvement in health decisions is prominent.

The IFSMT was selected as the guiding framework because it explicitly integrates individual, family, and contextual determinants of behavior, offering a more transparent structure than other classic conceptual models, such as the Social Cognitive Theory or socioecological models. IFSMT consists of three core propositions: (1) context factors including personal, family, and environmental conditions; (2) self-management process factors such as self-regulation, self-efficacy, and social facilitation; and (3) proximal and distal outcomes that influence clinical indicators and quality of life (32–34). This theoretical structure provides practical advantages for designing interventions that simultaneously strengthen self-regulation and family support in DMT2 patients.

Furthermore, IFSMT-based interventions are particularly relevant in low- and middle-income countries, where limited healthcare resources, strong family interdependence, and collectivistic cultural norms make family involvement central to chronic disease management (35, 36). Therefore, different, or even stronger, intervention effects may be expected compared with those in high-income countries, given the critical role of families in supporting daily self-care behaviors.

The IFSMT provides a comprehensive framework integrating individual, family, and contextual factors to support sustainable behavior change (32–34). Although IFSMT has been applied to various chronic conditions, empirical evidence regarding its use in DMT2, particularly in developing countries with diverse sociocultural contexts, remains limited (35, 36). Previous studies have primarily focused on improving knowledge or specific behavioral changes (37–39). However, although many studies have examined these outcomes individually or as secondary endpoints, comprehensive interventions that assess self-care, HbA1c control, and quality of life simultaneously, particularly with long-term follow-up, remain less common.

Addressing this gap is essential, as individual-focused interventions alone have shown limited long-term effectiveness (8, 40, 41). Strengthening family involvement may enhance patient self-regulation and support the continuity of self-care behaviors (42, 43). Therefore, this study aims to improve self-care, reduce HbA1c levels, and enhance the quality of life of patients with type 2 diabetes through a self-regulation intervention based on the Individual and Family Self-Management Theory (IFSMT).

## 2 Methods

### 2.1 Research design

This study used a quasi-experimental pretest–posttest control-group design. This design was chosen to evaluate the effectiveness of self-regulation modules based on Individual and Family Self-Management Theory (IFSMT) on self-care ability, glycemic control (HbA1c), and quality of life of patients with type 2 diabetes mellitus.

### 2.2 Sample and setting

This quasi-experimental study was conducted at a primary health care center in South Sulawesi, Indonesia, from March to May 2025. A purposive sampling method was used to recruit patients with type 2 diabetes mellitus who lived with their families and met the inclusion criteria. Power analysis using G*Power software (α = 0.05, power = 0.90, effect size = 0.8, number of groups = 2, number of measurements = 2) indicated that a minimum of 80 participants was required.

A total of 106 individuals were initially screened, and 16 were excluded due to incomplete baseline data. Thus, 90 eligible participants with complete data were included and subsequently randomized into the intervention group (n = 45) and the control group (n = 45). Group allocation was performed using a block-randomization method by independent researchers who were not involved in recruitment or data collection. Allocation confidentiality was maintained using opaque, sealed envelopes (SNOSE), which were opened only after written informed consent was obtained from each participant.

### 2.3 Inclusion and exclusion criteria

Inclusion criteria include: patients with a diagnosis of type 2 DM for at least 1 year, ≥18 years old, living with family, able to communicate in Indonesian, and willing to participate in the entire research series. Exclusion criteria include patients with severe complications, cognitive impairment, or those currently participating in another intervention program.

Exclusion criteria included: patients with severe complications, cognitive impairment, clinically diagnosed mental health disorders, high diabetes distress, or those currently participating in another intervention program. Severe complications were defined as advanced diabetes-related conditions that may limit study participation, such as diabetic foot ulcer grade ≥3, end-stage renal disease (CKD stage 4–5), recent stroke (<6 months), advanced heart failure (NYHA class III–IV), or uncontrolled proliferative retinopathy. Mental health disorders were defined as documented diagnoses of major depressive disorder, generalized anxiety disorder, bipolar disorder, or psychotic disorders based on medical records. Diabetes distress was screened using the Diabetes Distress Scale (DDS-17), with a cutoff score of ≥3 indicating high distress. Cognitive impairment was identified from existing clinical documentation, and participation in other intervention programs was confirmed through patient interviews and verification with clinic staff.

### 2.4 Intervention

The intervention in this study is a self-regulation program based on the Individual and Family Self-Management Theory (IFSMT), specifically designed to improve self-care skills, Glycemic control (HbA1c), and quality of life in patients with type 2 diabetes mellitus. The program is conducted over 12 weeks, with two sessions per week, each lasting 60–90 minutes in small groups of 8–10 participants.

The program is delivered by two trained diabetes nurse educators with more than five years of experience in diabetes care. Prior to the study, the educators completed a structured 8-hour training workshop covering the intervention protocol, communication strategies, family engagement techniques, and the use of all assessment tools. Training included role-play, supervised practice, and fidelity checks to ensure consistent delivery of the intervention.

The intervention module comprises four main topics: (1) Knowledge of diabetes self-care and lifestyle modification; (2) Self-regulation and stress management skills; (3) Family involvement and support; (3) Adaptation to long-term diabetes management. Each session combines interactive education, problem-solving discussions, and targeted exercises to strengthen patients’ self-regulation skills. Family members are encouraged to participate in selected sessions to reinforce family support.

The control group did not receive the IFSMT module intervention but received only a standard leaflet containing diabetes management information (dietary practices, physical activity, medication management, blood glucose monitoring, and foot care). Evaluations were conducted at baseline (pretest) and at the end of the intervention (week 12). The primary outcomes measured included self-care ability (assessed using standardized questionnaires), HbA1c levels (determined through laboratory examination), and quality of life (evaluated using the WHOQOL-BREF instrument).

### 2.5 Measurement tools

Some of the instruments used in this study, which were adopted and modified from previously validated measuring instruments, include:

#### 2.5.1 Compartments demographics

The researcher developed a questionnaire to collect data on respondents’ characteristics, including age, gender, education, occupation, duration of diabetes, and socio-economic status.

#### 2.5.2 Self-regulation module (based on IFSMT)

The intervention used a self-regulation module based on *Individual and Family Self-Management Theory (IFSMT).* This module contains structured training on diabetes self-care practices, including diet, medication management, blood sugar level monitoring, physical activity, foot care, and stress management. The module is designed to enhance the patient’s self-regulation capacity, thereby improving their quality of life.

#### 2.5.3 Self-care ability

- Knowledge: Measured using a modified Diabetes Knowledge Questionnaire-24 (DKQ-24) (44, 45), consisted of 15 items (8 favorable, 7 unfavorable) with True (1) and False (0) answer choices.
- Confidence: This includes self-efficacy, outcome expectations, and goal alignment, as measured by the modified Multidimensional Diabetes Questionnaire (MDQ) (33, 34, 46). Answers use a dichotomous scale: Yes (1) and No (0).
- Skills: Measured by the Summary of Diabetes Self-Care Activities (SDSCA) (47), consisting of 14 items related to diet, physical activity, blood sugar monitoring, medication management, and foot care. Scores range from 0 to 14; the higher the score, the better the self-care behavior.
- Stress Management: Assessed using the Perceived Stress Scale-10 (PSS-10) (48), consisting of 10 items (4 favorable and 6 unfavorable). The answers are coded as 0 or 1 based on the level of perceived stress.

#### 2.5.4 Quality of life

The quality of life was measured using the WHOQoL-BREF (49), a 15-question instrument that assesses four dimensions: physical health, psychological health, social relationships, and environment. Responses were evaluated using a Likert scale of 1–5, with higher scores indicating a better quality of life. The HbA1c value is used as an objective indicator for the physical health dimension.

### 2.6 Reliability and validity of study instruments

The content validity of the modified instruments was assessed by three expert judges: two community nursing specialists with more than 10 years of clinical and academic experience and one expert in research methodology. Experts were selected based on their clinical expertise, publication record, and prior involvement in instrument evaluation. Each judge independently evaluated item relevance, clarity, and cultural appropriateness using a 4-point relevance scale. Inter-judge agreement was assessed using the Item-Level Content Validity Index (I-CVI) and the Scale-Level CVI (S-CVI/Ave). All items achieved I-CVI values of 0.80-1.00, and the S-CVI/Ave was 0.93, indicating excellent content validity.

As several instruments were initially developed in English (DKQ-24, MDQ, SDSCA, PSS-10, and WHOQOL-BREF), a standardized cultural adaptation procedure was conducted. This process included forward translation by two bilingual nursing researchers, back-translation by an independent bilingual linguist, reconciliation by an expert panel, and pilot testing among 15 patients with characteristics similar to those of the study population. Minor wording adjustments were made to enhance clarity and cultural appropriateness.

Factorial validity was based on previously published psychometric studies, which have consistently supported the construct structures of these instruments (e.g., DKQ-24 two-factor model; SDSCA multidimensional model; WHOQOL-BREF four-domain model). Because the current study involved only minor modifications and the sample size was primarily powered for intervention testing rather than psychometric modeling, an independent factor analysis was not conducted.

Internal consistency reliability was assessed for each subscale of the self-care ability instrument and for the quality-of-life instrument. The self-care subscales demonstrated excellent reliability, with Cronbach’s alpha values of 0.958 (knowledge), 0.905 (confidence), 0.925 (skills), and 0.931 (stress management). Consistent with these results, McDonald’s Omega coefficients also indicated strong reliability (ω = 0.964, 0.913, 0.934, and 0.939, respectively).

The quality-of-life instrument similarly demonstrated high internal consistency (α = 0.920), as supported by a high McDonald’s Omega value (ω = 0.928). HbA1c was measured using standardized laboratory procedures; therefore, no reliability coefficient was applicable.

### 2.7 Ethical considerations

This research has received ethical approval from the Health Research Ethics Committee of Universitas Airlangga (No. 3382-KEPK). All prospective participants who met the criteria were provided with an explanation of the research’s objectives and procedures, and informed that they had the right to withdraw at any time without consequences. Written consent was obtained prior to data collection.

In accordance with ethical requirements, participants were explicitly informed that their existing clinical information including documentation related to cognitive impairment would be accessed for the purpose of determining eligibility for inclusion or exclusion in the study. The informed consent form clearly stated that the research team would review relevant clinical records as part of the screening process. Access to this clinical information was conducted only after ethical approval had been granted.

Participants’ identities were protected through the use of numerical codes on all questionnaires, with data access limited to the research team. Electronic data were stored in a password-protected personal computer, while printed documents were secured in locked cabinets. All research data will be stored for 5 years for academic purposes.

### 2.8 Data analysis

Respondent characteristic data were analyzed univariately, with results presented as frequency distributions (n) and percentage tables. Before analyzing the intervention’s effect, a characteristic equivalence test was conducted to compare the treatment and control groups. The equivalence test used the chi-square test for categorical data and the independent t-test for numerical data with a significance level of 0.05.

Before the primary analysis, the dataset was examined for missing values and potential outliers. Missing values were assessed using frequency checks, and the proportion of missing data across variables was less than 5%. Because the missing values were minimal and randomly distributed, a complete-case analysis (listwise deletion) was used. Outliers were evaluated using boxplots and standardized z-scores (|z| > 3.0), and no extreme outliers requiring removal were identified.

After ensuring data quality, the effect of the self-regulation module intervention on patients’ self-care ability and quality of life with type 2 diabetes mellitus was analyzed using the Wilcoxon Signed Rank Test and the Mann-Whitney U Test. Non-parametric tests were selected because several outcome variables did not meet the normality assumption, as assessed by the Shapiro–Wilk test, and their distributions were ordinal or skewed. The Wilcoxon Signed Rank Test was used to assess differences in paired ordinal data (pretest and posttest within the same group), while the Mann-Whitney U Test was used to assess differences between two independent groups (intervention and control). All analyses were conducted using IBM SPSS Statistics version 25.

### 2.9 Open science and transparency statement

This study was not preregistered because the research protocol had been finalized and data collection had begun prior to journal submission. Although preregistration was not completed, we sought to ensure transparency by providing detailed descriptions of the study protocol, intervention procedures, randomization, measurement tools, and analysis plan in the Methods section. No analytic codes were used, as the study employed standard statistical procedures in IBM SPSS Statistics version 25; however, the statistical output files and intervention materials (self-regulation module and implementation guide) are available from the corresponding author upon reasonable request. The absence of preregistration may introduce potential bias, but explicit reporting of the full protocol and analytic procedures helps to strengthen the study’s credibility and reproducibility.

## 3 Results

### 3.1 Demographic characteristics of the participants

The data in **Table 1** show that the majority of respondents in both groups were women (57.8% in the treatment group and 55.6% in the control group), with the most prevalent age group aged 46–55 years (35.6% and 37.8%). More than 70% of respondents had a history of diabetes mellitus, with more than half having suffered from it for ≥5 years. Most of them are educated in primary to secondary school, working (68.9% vs. 73.3%), and married (66.7% vs. 68.9%). The majority of respondents lived with their spouse and children (33.3% vs. 25.6%).

**Table 1:** Characteristics of patients with type 2 diabetes in the intervention and control groups (n = 90).

Characteristics	Intervention (n=45)		Control (n=45)		*p-value*
	Frequency	Percentage	Frequency	Percentage	
Gender					
Man	19	42.2	20	44.4	0.832^a^
Woman	26	57.8	25	55.6	
Age					
35-45 years old	15	33.3	20	44.4	0.160^a^
46-55 years old	16	35.6	8	17.8	
56-60 years old	14	31.1	17	37.8	
History of DM disease					
No history	12	26.7	9	20.0	0.618^a^
There is a history	33	73.3	36	80.0	
Socio-economic					
<2,500,000	19	42.2	23	51.1	0.526^a^
≥2.500.000	26	57.8	22	48.9	
Severity of the disease					
No complications	26	57.8	19	42.2	0.206^a^
There are complications	19	42.2	26	57.8	
Long suffering from DM (years)					
≥ 5 years	26	57.8	33	73.3	0.183^a^
< 5 years	19	42.2	12	26.7	
Education Level					
Not in school/Not finishing elementary school	5	11.1	4	8.9	0.533^a^
Primary school	21	46.7	16	35.6	
Junior high school	7	15.6	11	24.4	
High school	5	11.1	9	24.4	
College	7	15.6	5	11.1	
Work					
Not working	14	31.1	12	26.7	0.816^a^
Work	31	68.9	33	73.3	
Marital Status					
Unmarried	3	6.7	3	6.7	0.971^a^
Widow/Widower	12	26.7	11	24.4	
Married	30	66.7	31	68.9	
Living in the House					
Other children/families	16	35.6	20	44.4	0.458^a^
Husband/Wife	14	31.1	9	20.0	
Spouse and children	15	33.3	16	35.6	

a= uji chi-square.

The equality test revealed no significant differences in any demographic characteristics between the two groups (p > 0.05). Thus, the two groups were considered homogeneous, so differences in self-care abilities and quality of life were more likely to result from the intervention provided (**Table 1**).

The data in **Table 1** show that the majority of respondents in both groups were women (57.8% in the treatment group and 55.6% in the control group). The most prevalent age group was 46–55 years (35.6% and 37.8%). More than 70% of respondents had a history of diabetes mellitus, with more than half having lived with the condition for ≥5 years. The most common education level was primary to secondary school, and most participants were employed (68.9% vs. 73.3%) and married (66.7% vs. 68.9%). The most common living arrangement was living with a spouse and children (33.3% vs. 35.6%).

The homogeneity test showed no significant demographic differences between the two groups (p > 0.05). Therefore, both groups were considered comparable, and any differences in self-care abilities and quality of life were more likely attributable to the intervention (**Table 1**).

### 3.2 Self-care ability and quality of life among patients with type 2 diabetes mellitus in the intervention and control groups

Statistical analysis showed that the IFSMT-based self-regulation intervention produced significant improvements across all indicators of self-care ability in the intervention group. The Wilcoxon Signed Rank Test revealed significant increases in knowledge, confidence, skills, and stress management from pretest to posttest (p < 0.001 for all indicators). In contrast, the control group did not show significant changes in any self-care indicators (p > 0.05).

Although the control group had slightly lower pretest scores than the intervention group, this baseline difference was small and did not account for the improvements observed. The between-group comparisons using the Mann–Whitney U Test confirmed that posttest scores in the intervention group were significantly higher than those in the control group across all indicators of self-care ability (p < 0.001). This demonstrates that the observed improvements were attributable to the IFSMT-based self-regulation module rather than to baseline variation or natural progression.

Regarding HbA1c levels, the intervention group showed a statistically significant reduction (Wilcoxon test, p < 0.01), whereas the control group did not (p > 0.05). The Mann–Whitney U Test also showed that the reduction in HbA1c was significantly greater in the intervention group than in the control group (p < 0.05).

Quality-of-life outcomes also improved significantly following the intervention. The intervention group showed significant improvements in the psychological, social, and environmental domains (p < 0.01), while no significant improvements were observed in the control group (all p > 0.05). The Mann–Whitney U Test confirmed significant between-group differences in posttest scores (p < 0.05), indicating that the intervention meaningfully enhanced overall quality of life.

Collectively, these results demonstrate that the IFSMT-based self-regulation module was effective in improving self-care ability, reducing HbA1c levels, and enhancing quality of life among patients with type 2 diabetes mellitus, even after accounting for baseline differences between groups (**Table 2**).

**Table 2:** Distribution of self-care ability, quality of life, and HbA1c among patients with type 2 diabetes in the intervention and control groups (n = 90).

Variables and Indicators	Category	Intervention groups (n=45)		Control groups (n=45)	
		Pre test	Post test	Pre test	Post test
		Frequency (%)	Frequency (%)	Frequency (%)	Frequency (%)
Self-care ability					
Knowledge*	Less	22 (48.9)	2 (4.4)	17 (37.8)	15 (33.3)
	Enough	18 (40.0)	27 (60.0)	22 (48.9)	23 (51.1)
	Good	5 (11.1)	16 (35.6)	6 (13.3)	7 (15.6)
Confidence*	Less	29 (64.4)	1 (2.2)	27 (60.0)	26 (57.8)
	Enough	13 (28.9)	15 (33.3)	9 (20.0)	8 (17.8)
	Good	3 (6.7)	29 (64.4)	9 (20.0)	10 (24.4)
Skills*	Less	25 (55.6)	1 (2.2)	23 (51.1)	21 (46.7)
	Enough	16 (35.6)	21(46.7)	13 (28.9)	20 (44.4)
	Good	4 (8.9)	23 (51.1)	9 (20.0)	4 (8.9)
Management stress*	Heavy	22 (48.9)	0 (0.0)	19 (42.2)	16 (35.6)
	Keep	21 (46.7)	30 (66.7)	18 (40.0)	22 (48.9)
	Light	2 (4.4)	15 (33.3)	8 (17.8)	7 (15.6)
Quality of life					
Psychological*	Bad	10 (22.2)	2 (4.4)	15 (33.3)	13 (28.9)
	Keep	27 (60.0)	18 (40.0)	19 (42.2)	25 (55.6)
	Good	8 (17.8)	25 (55.6)	11 (24.4)	7 (15.6)
Relationship social*	Bad	11 (24.4)	2 (4.4)	10 (22.2)	4 (8.9)
	Keep	29 (64.4)	28 (62.2)	23 (51.1)	35 (77.8)
	Good	5 (11.1)	15 (33.3)	12 (26.7)	6 (13.3)
Environment*	Bad	4 (8.9)	1 (2.2)	5 (11.1)	4 (8.9)
	Keep	33 (73.3)	27 (60.0)	33 (73.3)	30 (66.7)
	Good	8 (17.8)	17 (37.8)	7 (15.6)	11 (24.4)
HbA1c*		Mean±SD	Mean±SD	Mean±SD	Mean±SD
		8.72 ± 2,23	7.40 ± 2.82	8.89 ± 1.98	9.03 ± 1.96

SD, Standard Deviation; HbA1c, %; *Significant (p<0.001).

### 3.3 Within-group changes in self-care ability, quality of life, and HbA1c following implementation of the IFSMT-based self-regulation module

Analysis using the Wilcoxon signed-rank test showed a significant increase in all domains of self-care ability in the intervention group (**Table 3**). In the domain of knowledge, the median score increased from 5.0 (IQR 4.0–8.0) to 9.0 (IQR 7.0–10.0) (Z = −5.29, p < 0.001), with a large effect size (r = −0.788) and a median difference of Δ = 3.0 (95% CI: 2.5–4.0). Domain belief showed an increase from 1.0 (IQR 1.0–2.0) to 3.0 (IQR 2.0–3.5) (Z = −5.72, p < 0.001; r = −0.853; Δ = 1.5, 95% CI: 1.0–2.0). Domain skills increased from 4.0 (IQR 3.0–6.0) to 8.0 (IQR 6.0–9.0) (Z = −5.51, p < 0.001; r = −0.821; Δ = 3.0, 95% CI: 2.5–3.5). The stress management domain increased from 4.0 (IQR 3.5–7.0) to 6.0 (IQR 4.0–7.0) (Z = −5.25, p < 0.001; r = −0.782; Δ = 2.5, 95% CI: 2.0–3.0). No significant changes were found in the four domains in the control group (p > 0.05).

**Table 3:** Within-group differences in self-care ability, quality of life, and HbA1c based on the wilcoxon signed-rank test in the intervention and control groups (n=90).

Variable	Group	Pre median (IQR)	Post median (IQR)	Z	p	Effect size (r)	Median difference (HL estimator) (95% CI)
Self-care ability:							
Knowledge	Intervention	5.0 (4.0-8.0)	9.0 (7.0-10.0)	−5.29	<0.001*	-0.788	Δ = 3.0 (95% CI: 2.5-4.0 )
	Control	8.0 (4.0-9.0)	8.0 (4.0-9.0)	−9.88	0.323	-0.147	Δ = 0.0 (95% CI: 0.0-0.5)
Belief	Intervention	1.0 (1.0-2.0)	3.0 (2.0-3.5)	−5.72	<0.001*	-0.853	Δ = 1.5 (95% CI: 1.0-2.0)
	Control	1.0 (1.0-2.0)	1.0 (1.0-2.5)	−3.55	0.737	-0.053	Δ = 0.0 (95% CI: -0.0-0.5)
Skills	Intervention	4.0 (3.0-6.0)	8.0 (6.0-9.0)	−5.51	<0.001*	-0.821	Δ = 3.0 (95% CI: 2.5-3.5)
	Control	4.0 (1.0-2.0)	5.0 (1.0-2.5)	−0.14	0.909	-0.017	Δ = 0.0 (95% CI: -1.0-1.5)
Stress management	Intervention	4.0 (3.5-7.0)	6.0 (4.0-7.0)	−5.25	<0.001*	-0.782	Δ = 2.5 (95% CI: 2.0-3.0)
	Control	5.0 (3.0-6.0)	5.0 (3.0-6.0)	−0.36	0.720	-0.053	Δ = 0.0 (95% CI: -0.5-0.5)
Quality of life:							
Psychological	Intervention	10.0 (9.0-12.0)	13.0 (7.0-10.0)	−5.29	<0.001*	-0.859	Δ = 3.5 (95% CI: 2.5-4.0)
	Control	10.0 (8.0-13.5)	11.0 (9.0-13.0)	−0.59	0.555	-0.088	Δ = 0.0 (95% CI: -0.5-1.0)
Relationship social	Intervention	7.0 (6.5-8.5)	10.0 (9.0-11.0)	−4.94	<0.001*	-0.736	Δ = 2.0 (95% CI: 1.5-3.0)
	Control	8.0 (7.0-11.0)	8.0 (8.0-10.0)	−1.68	0.092	-0.250	Δ = 0.5 (95% CI: 0.0-1.0)
Environment	Intervention	18.0 (16.5-20.0)	21.0 (19.0-22.0)	−4.59	<0.001*	-0.684	Δ = 2.0 (95% CI: 1.5-3.0)
	Control	18.0 (18.0-19.0)	19.0 (165-21.50)	−1.58	0.115	-0.236	Δ = 0.5 (95% CI: 0.0-1.0)
HbA1c	Intervention	8.40 (6.80-10.3)	7.30 (5.90-8.70)	−5.05	<0.001*	-0.752	Δ = -1.2 (95% CI: –1.6– –0.8)
	Control	8.60 (7.20-10.4)	8.50 (7.65-10.3)	−0.57	0.384	-0.084	Δ = 0.2 (95% CI:-0.2–0.5)

*=Significant; HbA1c= %; IQR=Interquartile Range.

Significant improvements were also observed in all domains of quality of life in the intervention group. The psychological domain increased from 10.0 (IQR 9.0–12.0) to 13.0 (IQR 7.0–10.0) (Z = −5.29, p < 0.001; r = −0.859; Δ = 3.5, 95% CI: 2.5–4.0). The social relationship domain increased from 7.0 (IQR 6.5–8.5) to 10.0 (IQR 9.0–11.0) (Z = −4.94, p < 0.001; r = −0.736; Δ = 2.0, 95% CI: 1.5–3.0). The domain environment increased from 18.0 (IQR 16.5–20.0) to 21.0 (IQR 19.0–22.0) (Z = −4.59, p < 0.001; r = −0.684; Δ = 2.0, 95% CI: 1.5–3.0). The control group showed no significant changes in all three domains (p > 0.05).

For clinical parameters, HbA1c levels in the intervention group decreased significantly from 8.40 (IQR 6.80–10.30) to 7.30 (IQR 5.90–8.70) (Z = −5.05, p < 0.001), with a large effect size (r = −0.752) and a median difference of Δ = −1.2 (95% CI: −1.6 to −0.8). No significant changes were found in the control group (p = 0.384).

Overall, **Table 3** shows that the intervention had a significant and consistent impact across domains of self-care ability, quality of life, and glycemic parameters, with effect sizes in the medium to large category, while the control group remained stable with no significant changes.

### 3.4 Between-group differences in self-care ability, quality of life, and HbA1c between intervention and control groups

As shown in **Table 4**, the intervention group demonstrated significantly greater post-intervention improvements across all domains of self-care ability than the control group. For the knowledge domain, the intervention group showed a significantly higher score (U = 620.5, Z = −3.193, p = 0.001, r = −0.337), with a median difference of 2 points (HL = 2; 95% CI: 1–3). Similar results were observed for belief (U = 405.0, Z = −5.062, p < 0.001, r = −0.533; HL = 1; 95% CI: 1–2), skills (U = 417.0, Z = −4.849, p < 0.001, r = −0.511; HL = 2; 95% CI: 2–3), and stress management (U = 490.5, Z = −4.322, p < 0.001, r = −0.456; HL = 2; 95% CI: 1–3).In contrast, the control group showed no significant changes across these domains.

**Table 4:** Within-group differences in self-care ability, qquality of life, and HbA1c based on the mann–whitney U test in the intervention and control groups (n=90).

Variabel	Group	Median (IQR)	U	Z	p-value	Effect size (r)	Median difference (HL estimator) (95% CI)
Self-care ability:							
Knowledge	Intervention	9.0 (7.0-10.0)	620.5	-3.193	0.001*	-0.337	Δ = 2.0 (95% CI: 1.0-3.0)
	Control	8.0 (4.0-9.0)					
Belief	Intervention	3.0 (2.0-3.5)	405.0	-5.062	<0.001*	-0.533	Δ = 1.0 (95% CI: 1.0-2.0)
	Control	1.0 (1.0-2.5)					
Skills	Intervention	8.0 (6.0-9.0)	417.0	-4.849	<0.001*	-0.511	Δ = 2.0 (95% CI: 2.0-3.0)
	Control	5.0 (4.0-7.0)					
Stress management	Intervention	6.0 (6.0-9.0)	490.5	-4.322	<0.001*	-0.456	Δ = 2.0 (95% CI: 1.0-3.0)
	Control	5.0 (3.0-6.0)					
Quality of life:							
Psychological	Intervention	13.0 (13.0-15.0)	460.0	-4.500	<0.001*	-0.474	Δ = 3.0 (95% CI: 2.0-4.0)
	Control	11.0 (9.0-13.0)					
Relationship social	Intervention	10.0 (9.0-11.0)	611.5	-3.287	0.001*	-0.346	Δ = 1.0 (95% CI: 1.0-2.0)
	Control	8.0 (8.0-10.0)					
Environment	Intervention	21.0 (19.0-22.0)	716.0	-2.411	0.016*	-0.254	Δ = 2.0 (95% CI: 0.0-3.0)
	Control	19.0 (16.5-21.5)					
HbA1c	Intervention	7.3 (5.9-8.7)	531.5	-3.884	<0.001*	-0.409	Δ = -1.7 (95% CI: -2.4--0.8)
	Control	8.5 (7.7-10.3)					

*=Significant; HbA1c= %; IQR=Interquartile Range.

Significant between-group differences were also found in quality-of-life indicators. Post-intervention psychological scores were significantly higher in the intervention group (U = 460.0, Z = −4.500, p < 0.001, r = −0.474; HL = 3; 95% CI: 2–4). Social relationship scores also differed significantly (U = 611.5, Z = −3.287, p = 0.001, r = −0.346; HL = 1; 95% CI: 1–2), as did environmental scores (U = 716.0, Z = −2.411, p = 0.016, r = −0.254; HL = 2; 95% CI: 0–3). The control group did not experience comparable improvements in any quality-of-life domain.

Regarding clinical outcomes, HbA1c levels showed a significant between-group difference following the intervention. The intervention group experienced a substantially greater reduction (U = 531.5, Z = −3.884, p < 0.001, r = −0.409), with a median decrease of −1.7% (HL = −1; 95% CI: −2.4 to −0.8), whereas the control group demonstrated no meaningful change.

Taken together, these findings indicate that the IFSMT-based self-regulation module led to significantly superior improvements in self-care ability, quality of life, and glycemic control compared with the control group, demonstrating the effectiveness of this intervention across behavioral and clinical outcomes.

## 4 Discussion

The results of this study demonstrate that the IFSMT-based self-regulation module effectively improved self-care abilities, quality of life, and glycemic control among patients with type 2 diabetes mellitus. These improvements were consistent across all measured domains and were supported by medium-to-large effect sizes and meaningful median differences reflected in the Hodges–Lehmann estimators. These findings provide strong evidence that the intervention produced clinically relevant changes beyond natural variation, reinforcing the theoretical assumptions of the Individual and Family Self-Management Theory (33, 34).

Improvements in knowledge, confidence, skills, and stress management support the central premise of IFSMT, which emphasizes the interaction between individual regulatory processes, family support, and contextual factors. Enhanced knowledge facilitates better health literacy, enabling patients to make informed self-care decisions, consistent with prior studies showing the role of structured diabetes education in achieving better self-management and glycemic outcomes (35). Increased confidence aligns with Bandura’s theory of self-efficacy, which posits that confidence is a key determinant of health behavior (50, 51). The improvements in self-care skills, particularly in diet, activity, and medication management, align with earlier findings linking behavioral competence to glycemic outcomes (52, 53). Similarly, improved stress management is associated with reduced diabetes distress, an important predictor of self-care engagement (54, 55).

The intervention also resulted in significant improvements across psychological, social, and environmental domains of quality of life. These findings are in line with prior work showing that better self-regulation and family involvement enhance emotional well-being and coping (56, 57), promote supportive social dynamics (58, 59), and facilitate better access to health services and adaptive functioning within the environment (60). The reduction in HbA1c observed in the intervention group confirms the clinical relevance of the approach, consistent with evidence that behavioral and educational interventions can improve glycemic control (61).

This study contributes to the literature by demonstrating that an IFSMT-based self-regulation module can serve as a comprehensive, theory-driven approach that integrates individual, family, and environmental components. Unlike traditional diabetes education that focuses primarily on knowledge transfer, this intervention emphasized self-regulatory skills, family engagement, and contextual adaptations, resulting in improvements that were both statistically significant and clinically meaningful.

## 5 Limitations

Several limitations should be acknowledged. First, although the instruments used demonstrated acceptable reliability, some psychometric limitations remain, particularly regarding cultural adaptation and reliance on self-report measures, which may introduce response bias. Second, the study was not preregistered in a public trial registry, which may limit methodological transparency and reproducibility. Third, the quasi-experimental design may be subject to residual confounding despite baseline equivalence. Fourth, the relatively short follow-up period limits conclusions about the sustainability of the improvements. Future studies should consider randomized controlled designs, preregistration, culturally validated measures, and long-term follow-up to strengthen the evidence base.

Overall, the findings support the IFSMT-based self-regulation module as an effective strategy for enhancing self-care behaviors, improving quality of life, and achieving better glycemic control. The integration of individual capacities with family and contextual support highlights its potential for implementation in clinical and community settings as a promotive and preventive approach in the management of type 2 diabetes mellitus.

## 6 Conclusions

This study demonstrates that self-regulation modules based on Individual and Family Self-Management Theory (IFSMT) are effective in enhancing self-care skills, reducing HbA1c levels, and improving the quality of life for patients with type 2 diabetes mellitus. This intervention strengthens knowledge, beliefs, skills, stress management, and family involvement to support patients’ self-care behaviors. These findings confirm that a theory-based educational approach that incorporates the role of the family can be an effective strategy for managing chronic diseases, especially type 2 diabetes mellitus.

Thus, IFSMT-based self-regulation modules have the potential to be integrated into nursing practice, both in primary and community health services, as preventive and promotive interventions to improve the effectiveness of diabetes management sustainably.

## Data Availability

The datasets generated and analyzed during the current study are not publicly available due to ethical and institutional restrictions. However, anonymized data can be made available from the corresponding author upon reasonable request. Requests to access the datasets should be directed to fadlietri@gmail.com.
